# The abdominal compartment syndrome: evolving concepts and future directions

**DOI:** 10.1186/s13054-015-0879-8

**Published:** 2015-05-06

**Authors:** Jan J De Waele, Manu LNG Malbrain, Andrew W Kirkpatrick

**Affiliations:** Department of Critical Care Medicine, Ghent University Hospital, De Pintelaan 185, Gent, 9000 Belgium; Intensive Care Unit and High Care Burn Unit, Ziekenhuis Netwerk Antwerpen, ZNA Stuivenberg, Lange Beeldekensstraat 267, Antwerpen 6, 2060 Belgium; Department of Surgery, Foothills Medical Centre, Calgary, AB T2N 2T9 Canada; Department of Critical Care Medicine, Foothills Medical Centre, Calgary, AB T2N 2T9 Canada; Regional Trauma Services, Foothills Medical Centre, Calgary, AB T2N 2T9 Canada

The modern-era abdominal compartment syndrome (ACS) was first described as a ‘new’ clinical entity in the 1980s in emergency surgery patients, despite being described over 100 years earlier [1]. This stimulated scientific research leading to a better understanding of ACS, and the development of strategies to prevent and treat the condition [2]. Simultaneously, several investigators described the impact of the prelude to ACS – intra-abdominal hypertension (IAH), where organ function is impaired at lower intra-abdominal pressure (IAP) in the range of 12–20 mmHg [3]. This new knowledge has improved our understanding of the interactions between the different body compartments and new pathophysiologic terms have been coined, such as the polycompartment syndrome [4]. Within this concept, abdominal wall compliance seems to be a key factor that has only recently been better investigated [5].

Although controversial initially, IAH is now widely accepted as a cause of organ dysfunction and ACS is recognized as a catastrophic disturbance of a patient’s physiology that requires urgent intervention and guided therapies [6]. Over the years, several strategies have been developed to attempt to mitigate IAH and to prevent progression to ACS [2]. IAP monitoring in patients at risk is the key element in early detection. IAP measurement methods are now universally available and have become safe, reliable and reproducible [7].

IAH does not only affect abdominal organs – raised IAP affects the different organ systems [6]; it greatly impacts the respiratory system, hemodynamics, and even cerebral perfusion. IAH is an important determinant of the compliance of the respiratory system, and practical consequences for mechanically ventilated patients are important [8]. IAH influences our traditional filling pressures, and volumetric preload indices better reflect the true preload status in IAH [9].

Improved insights into the pathophysiology and causes of IAH and ACS have led to improved management of patients at risk [10]. Open abdomen management and parallel changes in resuscitation strategies have now dramatically reduced the incidence of full-blown ACS [11], which has been observed most dramatically in trauma patients [12]. This integrated, IAH-focused approach has almost completely abolished ACS in some hospitals [13].

Whereas the incidence of end-stage, highly lethal, overt ACS is decreasing, IAH persists and is likely to increasingly do so as critically ill patients increasingly survive initial insults. Better understanding of the risks associated with IAH is thus necessary, as well as recognizing clinically important thresholds [14] and critically assessing the impact of different interventions aimed at IAP.

One of the major challenges currently in our ICUs is the management of open abdomen patients. An open abdomen is at high risk of a myriad of complications and planning for a safe same-admission closure begins immediately after opening [15]. It has become clear that ICU management also impacts the feasibility of closure, and more information is needed on how intensivists and surgeons can collaborate to reach this goal. Avoiding massive fluid overload and initiating de-resuscitation as soon as possible should be considered [12].

As a highly focused specialist society, the World Society of the Abdominal Compartment Syndrome (WSACS) has been assisting healthcare workers to better understand IAH and ACS; the WSACS’s efforts have certainly contributed to many advances that have been made in the past [2]. As ACS is no longer the main challenge in this context, the WSACS recently changed name to the WSACS – the Abdominal Compartment Society in order to maintain relevance and concordance between the priorities of the society and the reprioritization in the challenges that are ahead of us. WSACS – the Abdominal Compartment Society is thus ready to address the challenges of truly appreciating the physiology, pathophysiology, and reconstructive anatomy of the abdominal compartment within the overall context of human injury and illness.

WSACS – the Abdominal Compartment Society remains a dedicated multidisciplinary international society of clinicians, scientists, clinician-scientists, and other healthcare workers dedicated to understanding the holistic implications of IAP. Further, the society understands and promotes health through championing anatomically functional abdominal compartment reconstruction at the earliest time after critical illness/injury, balancing the needs at all times for physiologic decompression and avoidance of IAH with anatomic reconstruction.

Understanding the subtle implications of modest IAH on all critically ill/injured patients and especially validating potential therapeutic interventions with new sound evidence remain our biggest challenges. A new emphasis will be placed upon embracing those engaged in the emerging surgical subspecialty of abdominal wall reconstruction. Ultimately, complete integration of these findings in the management of patients with relevant abdominal conditions affected by IAH remains the final goal.

In conclusion, IAH and ACS have evolved from poorly understood and inconsistently diagnosed disorders. Overall, ACS is decreasing through early recognition and directed management of IAH. In this context, WSACS – the Abdominal Compartment Society will continue its educational activities, support research, and promote evidence-based guidelines in order to continue to improve outcome in critically ill patients.

## Abbreviations

ACS: Abdominal compartment syndrome
IAH: Intra-abdominal hypertension
IAP: Intra-abdominal pressure
WSACS: World society of the abdominal compartment syndrome

## References

[1] Van Hee R (2007). Historical highlights in concept and treatment of abdominal compartment syndrome. Acta Clin Belg Suppl..

[2] Kirkpatrick AW, Roberts DJ, De Waele J, Jaeschke R, Malbrain ML, De Keulenaer B (2013). Intra-abdominal hypertension and the abdominal compartment syndrome: updated consensus definitions and clinical practice guidelines from the World Society of the Abdominal Compartment Syndrome. Intensive Care Med..

[3] Kirkpatrick AW, Roberts DJ, De Waele J, Laupland K (2014). Is intra-abdominal hypertension a missing factor that drives multiple organ dysfunction syndrome?. Crit Care..

[4] Malbrain ML, Roberts DJ, Sugrue M, De Keulenaer BL, Ivatury R, Pelosi P (2014). The polycompartment syndrome: a concise state-of-the-art review. Anaesthesiol Intensive Ther..

[5] Malbrain ML, De Laet I, De Waele JJ, Sugrue M, Schachtrupp A, Duchesne J (2014). The role of abdominal compliance, the neglected parameter in critically ill patients – a consensus review of 16. Part 2: measurement techniques and management recommendations. Anaesthesiol Intensive Ther.

[6] De Waele JJ, De Laet I, Kirkpatrick AW, Hoste E (2011). Intra-abdominal hypertension and abdominal compartment syndrome. Am J Kidney Dis..

[7] De Waele JJ, De Laet I, Malbrain ML (2007). Rational intraabdominal pressure monitoring: how to do it?. Acta Clin Belg..

[8] Pelosi P, Vargas M (2012). Mechanical ventilation and intra-abdominal hypertension: ‘Beyond Good and Evil’. Crit Care..

[9] Cheatham ML, Malbrain ML (2007). Cardiovascular implications of abdominal compartment syndrome. Acta Clin Belg Suppl..

[10] Holodinsky JK, Roberts DJ, Ball CG, Reintam Blaser A, Starkopf J, Zygun DA (2013). Risk factors for intra-abdominal hypertension and abdominal compartment syndrome among adult intensive care unit patients: a systematic review and meta-analysis. Crit Care..

[11] Mentula P, Leppaniemi A (2010). Prophylactic open abdomen in patients with postoperative intra-abdominal hypertension. Crit Care..

[12] Duchesne JC, Kaplan LJ, Balogh ZJ, Malbrain ML. Role of permissive hypotension, hypertonic resuscitation and the global increased permeability syndrome in patients with severe hemorrhage: adjuncts to damage control resuscitation to prevent intra-abdominal hypertension. Anaesthesiol Intensive Ther. 2014; doi:10.5603/AIT.a2014.0052. [Epub ahead of print]. 10.5603/AIT.a2014.0052 25293626

[13] Balogh ZJ, Martin A, van Wessem KP, King KL, Mackay P, Havill K (2011). Mission to eliminate postinjury abdominal compartment syndrome. Arch Surg..

[14] Smit M, Hofker HS, Leuvenink HG, Krikke C, Jongman RM, Zijlstra JG (2013). A human model of intra-abdominal hypertension: even slightly elevated pressures lead to increased acute systemic inflammation and signs of acute kidney injury. Crit Care..

[15] Demetriades D, Salim A (2014). Management of the open abdomen. Surg Clin North Am..

